# 

**Published:** 1871-09

**Authors:** 


### Contents of September Number.

### ORIGINAL DEPARTMENT.

ART. I —Rhinoplasty—Talicotian Operation. Result illustrated. Clinical Lecture by Prof. J. F. Miner. Reported by W. W. Miner, member of the class, 1870-71 ..... 41
ART. II.—A New method of treating Chronic Alcoholism by Chloral Hydrate and Bromide of Potassium. By F. Bradnack, M. D ..... 44
ART. III.—Hospital Notes. By C. C. F. Gay, M. D ..... 47

### CORRESPONDENCE.

Quackery, and the National system of Medical Education. By H. R. Hopkins, M. D ..... 50
Homœopathy and Regular Medicine. Reply by E. V. Stoddard, M. D ..... 53

### MISCELLANEOUS.

The Diagnostic and Prognostic value of Heart-Sounds. By Austin Flint, M. D ..... 54
Dangerous and fatal results from the use of Hydrate of Chloral. By H. W. Fuller, M. D ..... 57
Cundurango ..... 60
Wanted—A Rara Avis ..... 62
Action of Mercury on the Liver ..... 63
On the application of Carbolic Acid as a local Anæsthetic in Surgical Operations ..... 63
Deaths from Chloral Hydrate ..... 65
Dose of Chloral ..... 66
The source of Scarlet Fever Poison ..... 66
The formation of Urea ..... 67
Ulcers of the Cervix Uteri ..... 67
The Hypodermic use of Sulphate of Quinia, by Francis L. Haynes, M. D ..... 68
The best period of operating for Laceration of the Perineum ..... 70
In an Obstetrical case, should the physician take his Forceps with him? ..... 71

### EDITORIAL DEPARTMENT.

Cundurango—Answer to Correspondents ..... 73
Regular Medicine and Homœopathy—Remark ..... 74
Appointment ..... 75
Post-Mortem Poisoning ..... 75
An Abortionist's "Diploma" ..... 75

BOOKS REVIEW:

On Syphilitic Epilepsy, by Reuben A. Vance, M. D ..... 76
Plastics and Orthopedics, by David Prince, M. D ..... 76
The late Dr. Conolly, of Hanwell, England, by Charles A. Lee, M. D ..... 76
Artificial Induction of Labor in Uræmia, by Samuel C. Busey, M. D ..... 76
Meeting of the 22d Annual Meeting of the American Medical Association ..... 77
Transaction of the Minnesota State Medical Society ..... 77
A treatise on the Nervous System, by William A. Hammond, M. D ..... 77
Opium and Opium Appetite, by Alonzo Calkins, M. D ..... 78
Tanner on Infancy and Childhood, by Meadows ..... 78

Death of Thomas Hawkes Tanner ..... 79
A successful method af tteating certain cases of Dysmenorrhœa and Sterility ..... 79
The Future State of Doctors ..... 79

BOOKS AND PAMPHLETS RECEIVED ..... 80

